# Supplementary material for: Innate immune and proinflammatory signals activate the Hippo pathway via a Tak1-STRIPAK-Tao axis
Source: Nat Commun. 2024 Jan 2;15:145. doi: 10.1038/s41467-023-44542-y (PMC10761881; doi:10.1038/s41467-023-44542-y)
Supplement: Supplementary file 1 — Supplementary Information [file 41467_2023_44542_MOESM1_ESM.pdf]

Supplementary Information for

Innate immune and proinflammatory signals activate the Hippo pathway via a Tak1-STRIPAK-Tao axis

Yinan Yang[1#], Huijing Zhou[1#], Xiawei Huang[1#], Chengfang Wu[1], Kewei Zheng[1], Jingrong Deng[1], Yonggang Zheng[2], Jiahui Wang[1], Xiaofeng Chi[1], Xianjue Ma[3], Huimin Pan[1], Rui Shen[1], Duojia Pan[2], Bo Liu[1,*]

1. State Key Laboratory of Cellular Stress Biology, Innovation Center for Cell Signaling Network, School of Life Sciences, Xiamen University, Xiamen, Fujian, 361102, China.

2. Department of Physiology, Howard Hughes Medical Institute, University of Texas Southwestern Medical Center, Dallas, Texas 75390, USA.

3. Westlake Laboratory of Life Sciences and Biomedicine, Hangzhou, Zhejiang, 310024, China.

# Equally Contributing Authors

* Corresponding author: bliu23@xmu.edu.cn

This PDF file includes:

- Supplementary Figures 1-9
- Supplementary Table 1

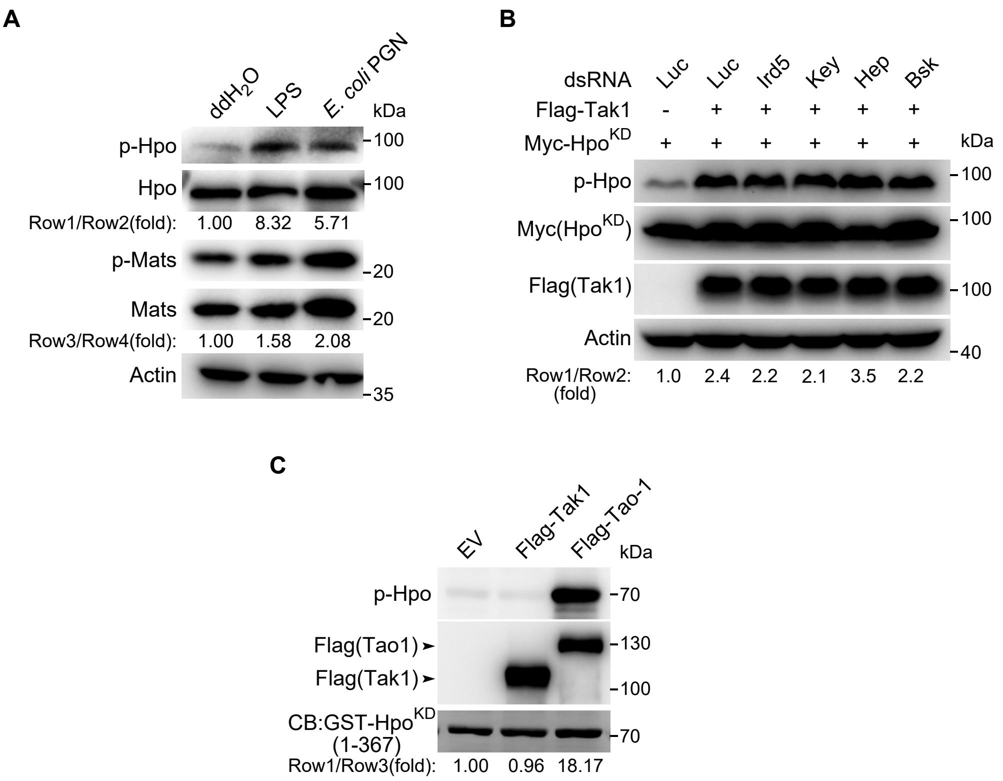

Supplementary Fig. 1: Tak1 induces the phosphorylation of Hippo pathway components.

(A) *Drosophila* S2 cells were pretreated with 20-Hydroxyecdysone (1 μM, 48 h) before treated with LPS (10 μg/mL, 10 min) or *E. coli* PGN (20 μg/mL, 10 min). Note the increased phosphorylation of Hpo or Mats upon LPS or PGN treatment.

(B) S2R+ cells were pre-treated with dsRNAs of indicated genes before transfected with indicated plasmids. Western blot was performed to monitor the phosphorylation of Hpo. A kinase-dead form of Hpo (Hpo^{KD}) was used to avoid autophosphorylation of Hpo. Note that the enhanced phosphorylation of Hpo upon Tak1 co-expression was unaltered by Ird5, Key, Hep or Bsk knockdown.

(C) *In vitro* kinase assay was performed using bacterially purified GST-Hpo^{KD} (kinase dead form of Hpo) and Tak1 or Tao-1 immunoprecipitated from S2R+ cells. Note the increased Hpo phosphorylation by Tao-1, but not Tak1. EV, empty vector; CB, Coomassie blue.

Data shown are representative of at least three independent experiments. Source data are provided as a Source Data file.

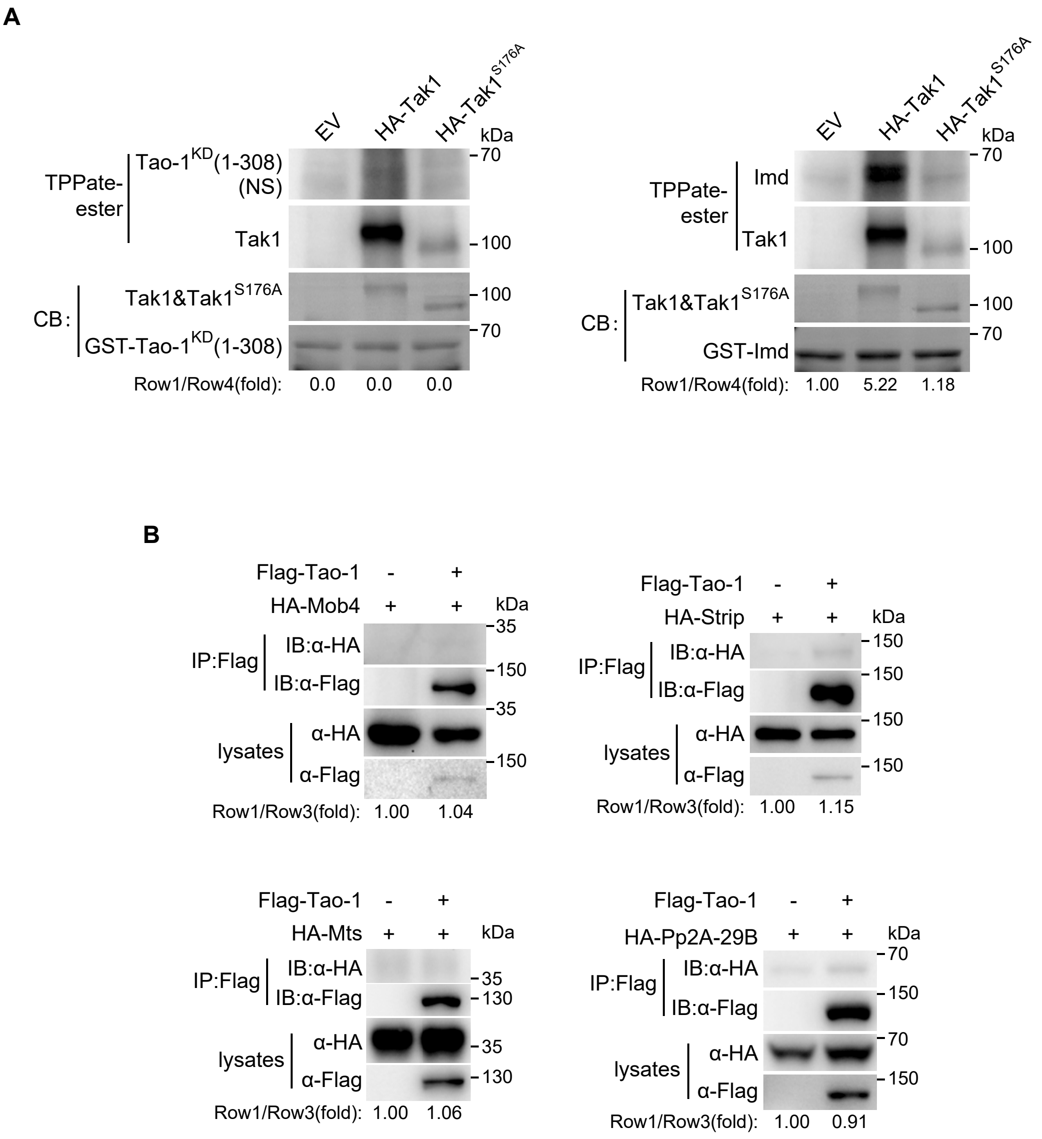

Supplementary Fig. 2: The phosphorylation of Tao-1 is negatively regulated by STRIPAK PP2A complex.

(A) *In vitro* kinase assay was performed using bacterially purified GST-Tao-1^{KD} (1-308) (left panel) or GST-Imd (right panel) and Tak1 or Tak1^{S176A} immunoprecipitated from S2R+ cells. Note the unaltered thiophosphate-ester signals on Tao-1 after incubating with either Tak1 or Tak1^{S176A} (left panel); the increased thiophosphate-ester signals on Imd after incubating with Tak1, but not Tak1^{S176A} (right panel). Also note the thiophosphate-ester signals on Tak1 (due to autophosphorylation) in both panels. EV, empty vector; CB, Coomassie blue; TPPate, thiophosphate. NS, non-specific.

(B) Immunoprecipitation was performed in S2R+ cells transfected with indicated plasmids. Note no interaction between Tao-1 and Mob4, Strip, Mts or Pp2A-29B.

Data shown are representative of at least three independent experiments. Source data are provided as a Source Data file.

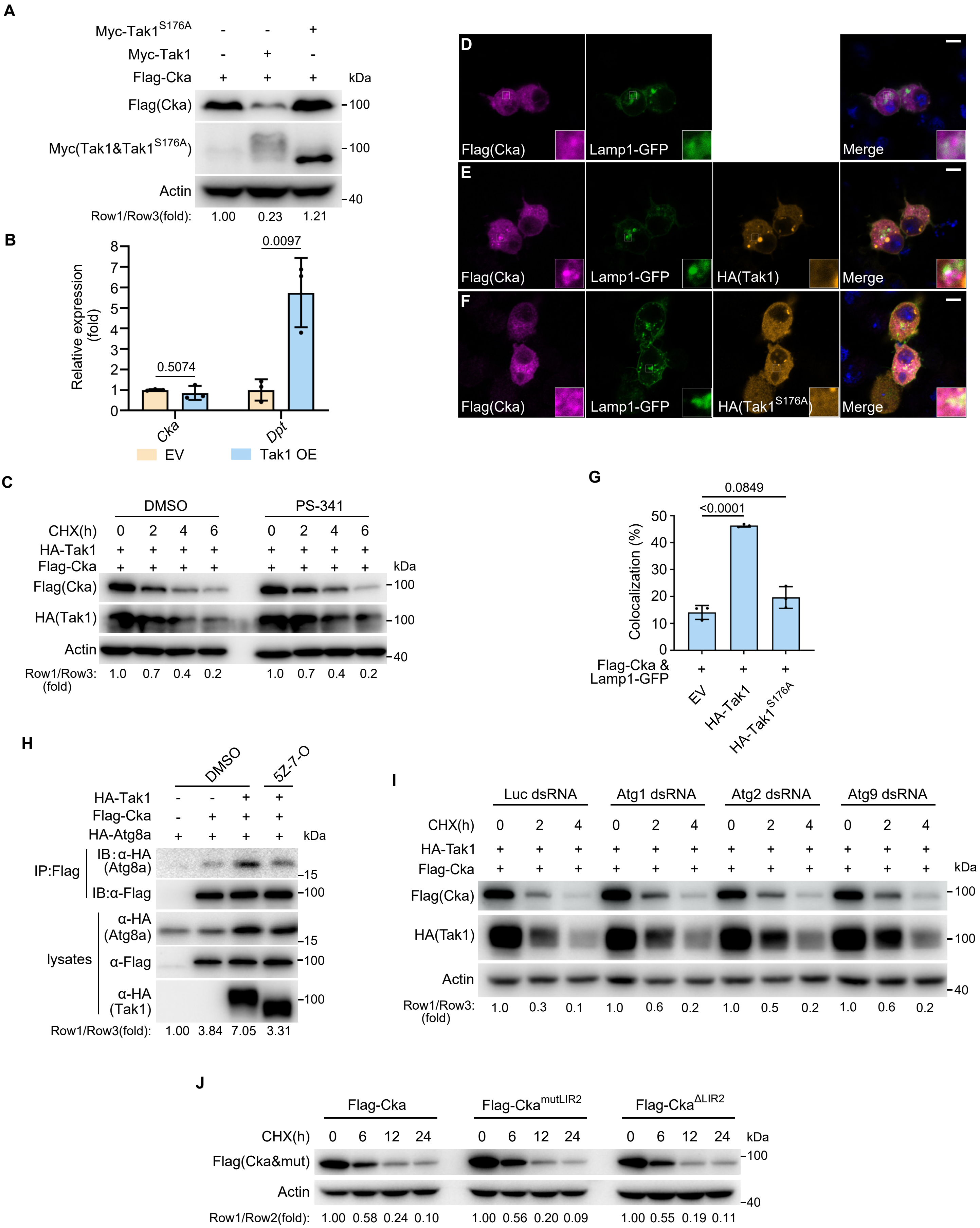
A
Myc-Tak1S176A - - +
Myc-Tak1 - + -
Flag-Cka + + +
kDa
Flag(Cka) 100
Myc(Tak1&Tak1S176A) 100
Actin 40
Row1/Row3(fold): 1.00 0.23 1.21
B
Relative expression (fold)
0.5074
0.0097
Cka
Dpt
EV
Tak1 OE
C
DMSO
PS-341
CHX(h) 0 2 4 6 0 2 4 6
HA-Tak1
Flag-Cka
Flag(Cka) 100
HA(Tak1) 100
Actin 40
Row1/Row3: (fold) 1.0 0.7 0.4 0.2 1.0 0.7 0.4 0.2
D
E
F
Flag(Cka)
Lamp1-GFP
HA(Tak1)
HA(Tak1S176A)
Merge
G
Colocalization (%)
0.0849
<0.0001
Flag-Cka & Lamp1-GFP
EV
HA-Tak1
HA-Tak1S176A
H
DMSO
5Z-7-O
HA-Tak1 - - + +
Flag-Cka - + + +
HA-Atg8a + + + +
kDa
IP:Flag
IB:α-HA (Atg8a) 15
IB:α-Flag 100
lysates
α-HA (Atg8a) 15
α-Flag 100
α-HA (Tak1) 100
Row1/Row3(fold): 1.00 3.84 7.05 3.31
I
Luc dsRNA
Atg1 dsRNA
Atg2 dsRNA
Atg9 dsRNA
CHX(h) 0 2 4 0 2 4 0 2 4 0 2 4
HA-Tak1
Flag-Cka
kDa
Flag(Cka) 100
HA(Tak1) 100
Actin 40
Row1/Row3: (fold) 1.0 0.3 0.1 1.0 0.6 0.2 1.0 0.5 0.2 1.0 0.6 0.2
J
Flag-Cka
Flag-CkamutLIR2
Flag-CkaΔLIR2
CHX(h) 0 6 12 24 0 6 12 24 0 6 12 24
kDa
Flag(Cka&mut) 100
Actin 40
Row1/Row2(fold): 1.00 0.58 0.24 0.10 1.00 0.56 0.20 0.09 1.00 0.55 0.19 0.11

Supplementary Fig. 3: Tak1 induces the lysosomal degradation of Cka.

(A) S2R+ cells were transfected with indicated plasmids. Note the reduced protein level of Cka upon co-expression with Tak1, but not Tak1^{S176A}.

(B) S2R+ cells were transfected with empty vector (EV) or Tak1 plasmid. qRT-PCR was performed to monitor the mRNA level of *Cka* or *Dpt*. Data are mean ± s.d., n=3 biological replicates, two-tailed Student's *t*-test.

(C) S2R+ cells transfected with indicated plasmids were treated with CHX (50 μg/mL) together with DMSO or PS-341 (0.5 μM) for indicated times. Note that Tak1-induced Cka degradation was not affected by PS-341 treatment.

(D-G) Immunostaining showing enhanced colocalization of Cka (purple) and LAMP1 (green) upon co-expression of Tak1, but not Tak1^{S176A} (orange) (D-F). Quantification of Cka and LAMP1 colocalization is shown in (G). Data in (G) were analyzed using two-tailed Student's *t*-test and presented as mean ± s.d., n=3 biological replicates. Scale bars, 4 μm; EV, empty vector.

(H) Immunoprecipitation was performed in S2R+ cells transfected with indicated plasmids and treated with DMSO or 5Z-7-Oxozeaenol (2 μM, 3 h). Note that the enhanced Cka-Atg8a association resulted from Tak1 co-expression was inhibited by 5Z-7-Oxozeaenol treatment.

(I) S2R+ cells pretreated with dsRNAs of Luciferase or several Atg proteins and then transfected with indicated plasmids were treated with CHX (50 μg/mL) for indicated times. Note that the accelerated degradation of Cka upon Tak1 co-expression was impeded by Atg1, Atg2 or Atg9 knockdown.

(J) S2R+ cells were transfected with indicated plasmids and treated with CHX (50 μg/mL) for designated times. Note the comparable degradation kinetics of Cka, Cka^{mutLIR2} and Cka$^{\Delta LIR2}$.

Data shown are representative of at least three independent experiments. Source data are provided as a Source Data file.

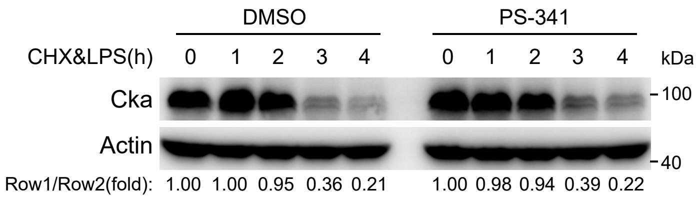

Supplementary Fig. 4: LPS-induced degradation of endogenous Cka is not through proteasomal pathway.

Drosophila S2 cells were pretreated with 20-Hydroxyecdysone (1 μM, 48 h) to potentiate the immune response before treated with CHX and LPS together with DMSO or PS-341 (0.5 μM). Note that the degradation of endogenous Cka resulted from LPS treatment was not affected by PS-341 treatment.

Data shown are representative of at least three independent experiments. Source data are provided as a Source Data file.

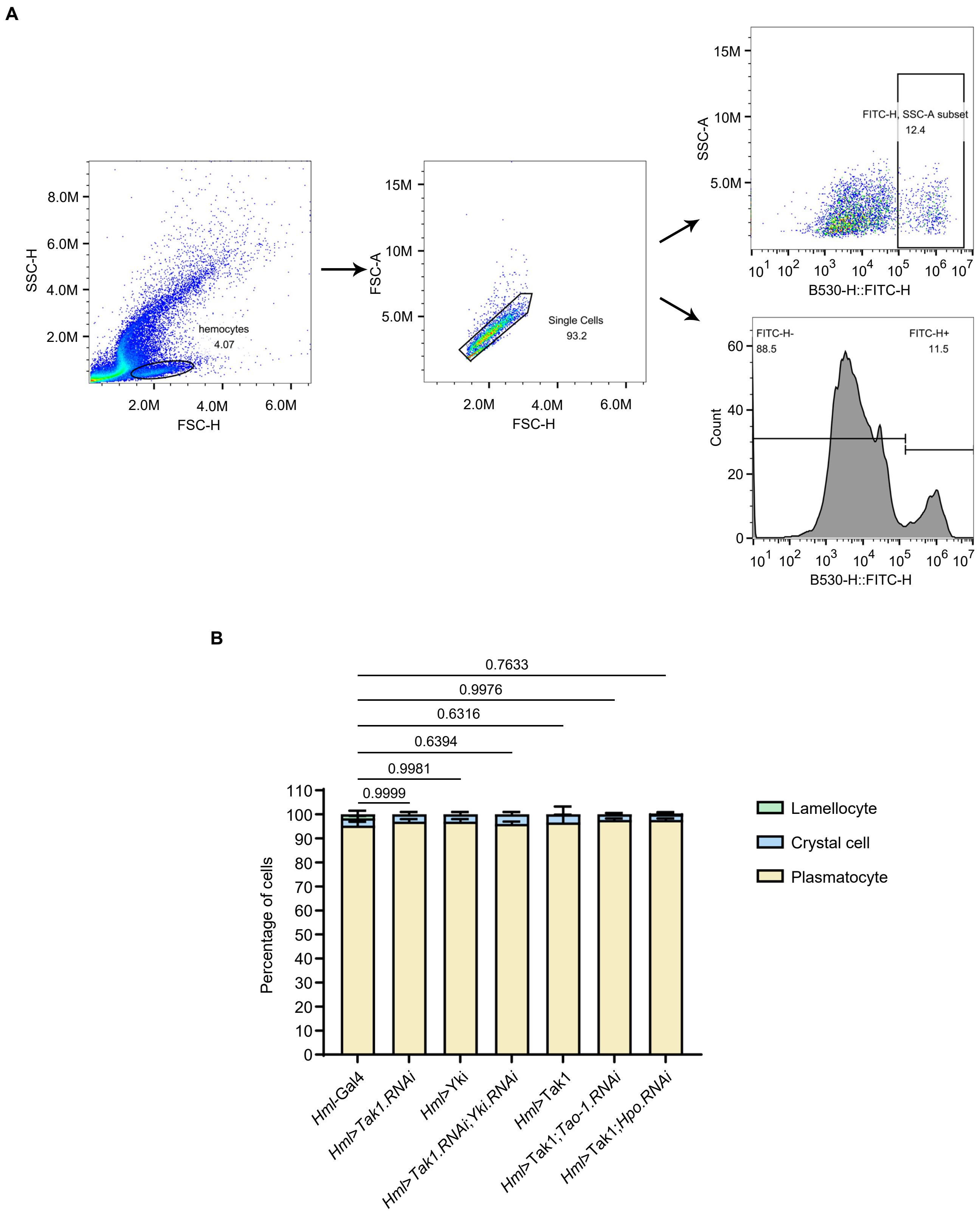

Supplementary Fig. 5: Tak1-mediated Hippo signaling regulates the phagocytic activity of hemocytes.

(A) The scheme illustrates the gating strategy for the phagocytosis assay (Fig. 5A-E). (B) Proportion of different hemocytes in fly larvae. The percentage of plasmatocytes, crystal cells and lamellocytes were shown as 100% stacked bar chart. Note the comparable proportion of plasmatocytes in the larvae of indicated genotype. The

percentage of the plasmatocyte was analyzed using two-way ANOVA with Dunnett's multiple comparisons test and shown as mean + s.d., *n*=3 biological replicates. Source data are provided as a Source Data file.

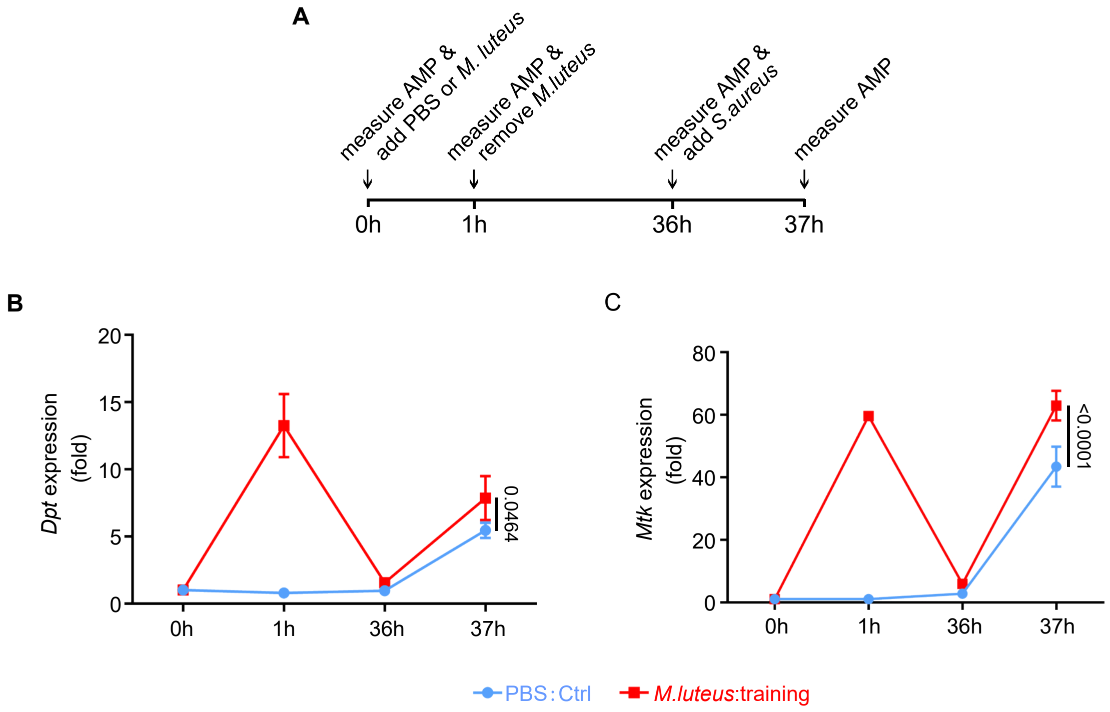

Supplementary Fig. 6: Bacterial combination of *M. luteus* and *S. aureus* elicits trained immunity feature in S2 cells.

(A) Schematic of S2 cell treatment.

(B-C) mRNA of *Dpt or Mtk* was monitored by qRT-PCR at indicated times after treatment. Data shown are mean ± s.d., *n*=3 biological replicates, two-way ANOVA, Sidak's multiple comparisons test. Source data are provided as a Source Data file.

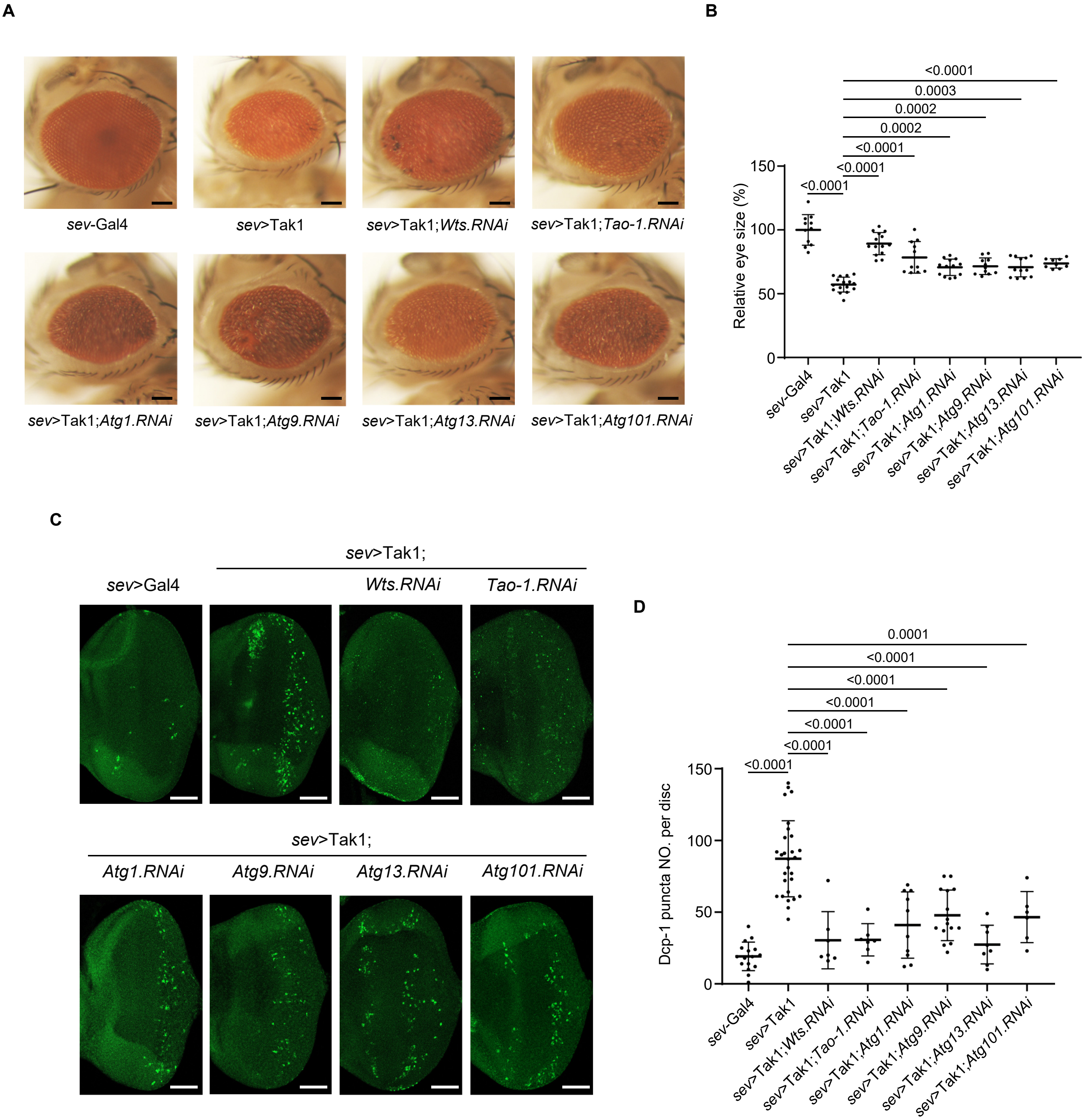

Supplementary Fig. 7: Hippo signaling is involved in Tak1-induced tissue ablation.

(A) Adult eye images of the indicated genotypes, all taken under the same magnification. Note that the reduced eye size resulted from Tak1 overexpression was partially rescued by simultaneous depletion of Tao-1, Wts, Atg1, Atg9, Atg13 or Atg101. Scale bars, 100 μm.

(B) Quantification of eye size in (A). Data shown are mean ± s.d., (*sev*-Gal4: *n*=11 flies; *sev*>Tak1: *n*=16 flies; *sev*>Tak1;*wts.RNAi*: *n*=14 flies; *sev*>Tak1;*Tao-1.RNAi*: *n*=11 flies; *sev*>Tak1;*Atg1.RNAi*: *n*=13 flies; *sev*>Tak1;*Atg9.RNAi*: *n*=11 flies; *sev*>Tak1;*Atg13.RNAi*: *n*=12 flies; *sev*>Tak1;*Atg101.RNAi*: *n*=9 flies), two-tailed Student's *t*-test.

(C) Eye discs dissected from 3rd instar larvae with indicated genotype were stained for cleaved Dcp-1 (green). Note that the increased number of cleaved Dcp-1 puncta from Tak1 overexpression was partially suppressed by simultaneous depletion of Tao-1, Wts, Atg1, Atg9, Atg13 or Atg101. Scale bars, 50 μm.

(D) Quantification of cleaved Dcp-1 puncta number in (C). Data shown are mean ± s.d., (*sev*-Gal4: *n*=16 eye discs; *sev*>Tak1: *n*=28 eye discs; *sev*>Tak1;*wts.RNAi*: *n*=7 eye discs; *sev*>Tak1;*Tao-1.RNAi*: *n*=7 eye discs; *sev*>Tak1;*Atg1.RNAi*: *n*=10 eye discs; *sev*>Tak1;*Atg9.RNAi*: *n*=15 eye discs; *sev*>Tak1;*Atg13.RNAi*: *n*=8 eye discs; *sev*>Tak1;*Atg101.RNAi*: *n*=6 eye discs), two-tailed Student's *t*-test.

Data shown are representative of at least three independent experiments. Source data are provided as a Source Data file.

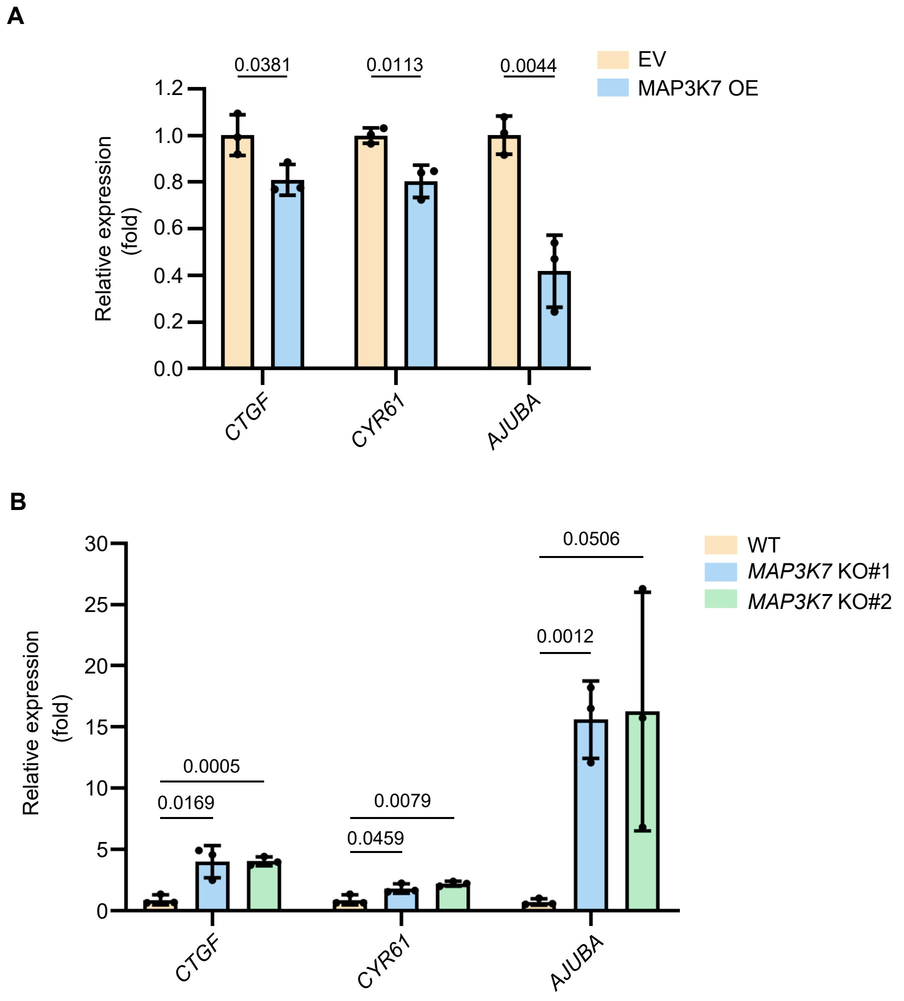

Supplementary Fig. 8: MAP3K7 regulates the Hippo signaling activity. qRT-PCR assay showing that mRNA level of YAP target genes was reduced by MAP3K7 overexpression (A), while was heightened by *MAP3K7* knockout (B). Data were analyzed using two-tailed Student's t-test and presented as mean ± s.d., *n*=3 biological replicates. EV, empty vector. Source data are provided as a Source Data file.

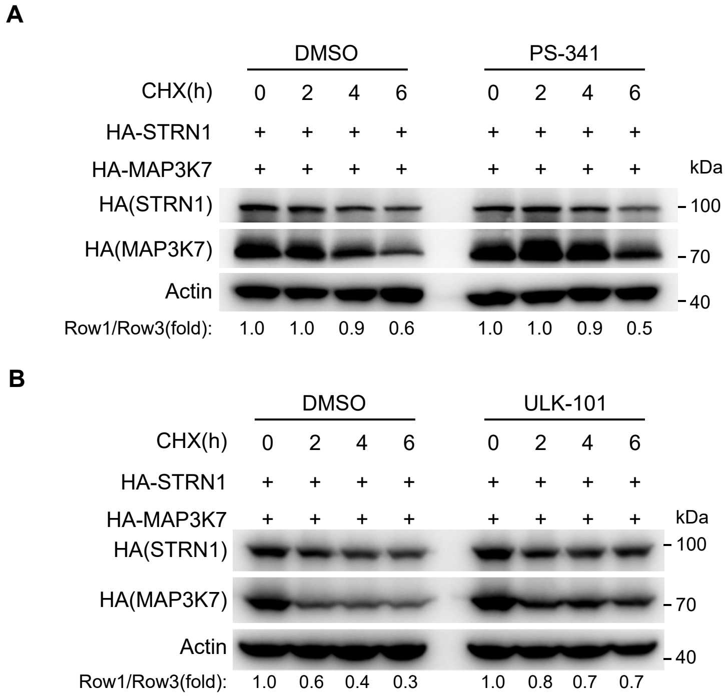

Supplementary Fig. 9: MAP3K7-induced degradation of STRN1 is not through proteasomal pathway, but autophagy-lysosomal pathway.

(A) HEK293T cells transfected with indicated plasmids were treated with CHX (50 μg/mL) together with DMSO or PS-341 (0.5 μM) for indicated times. Note that MAP3K7-induced STRN1 degradation was not affected by PS-341 treatment.

(B) Similar to (A) except that the cells were pre-treated with ULK-101 (5 μM) for 1 h before CHX treatment. Note that MAP3K7-induced STRN1 degradation was suppressed by ULK-101 treatment.

Data shown are representative of at least three independent experiments. Source data are provided as a Source Data file.

Supplementary Table 1: Knockdown efficiency of the RNAi in this study

**Gene**	**Knockdown efficiency (s.d.)**	**Related figure**
Hpo	58.38 (4.96)	1E
	75.89 (5.47)	5E, 6E, S5, 6F
Wts	51.22 (10.47)	1E
	34.45 (2.73)	S7
Yki	67.11 (13.46)	5C, 6C, S5, 6F
Tao-1	47.38 (7.78)	1G, 1I
	31.32 (6.12)	5E, 6D, S5, 6F
	57.13 (3.56)	S7
Slmap	57.01 (14.29)	2A, 2F, 2G
Fgop2	50.79 (3.01)	2A, 2E, 2G
Cka	68.49 (10.51)	2A, 2E, 2F
Mob4	91.98 (8.49)	2A
Strip	90.97 (1.43)	2A
Mts	73.34 (8.92)	2A
Pp2A-29B	20.80 (1.82)	2A
Ird5	68.94 (12.45)	S1B
Key	43.94 (4.22)	S1B
Hep	59.92 (3.82)	S1B
Bsk	49.57 (18.21)	S1B
Tak1	71.38 (7.12)	1K
	24.32 (5.92)	5A, 5C, 6A, 6C, S5, 6F
Atg1	68.12 (6.82)	S3I
	50.51 (3.43)	S7
	48.72 (5.59)	4F
Atg2	95.31(1.82)	S3I
Atg8a	62. 26 (3.43)	3K
Atg9	95.45 (4.21)	S3I
	39.68 (4.59)	S7
	46.78 (4.85)	4F
Atg13	69.75 (3.03)	S7
	72.68 (8.49)	4F
Atg101	55.36 (3.24)	S7
	59.14 (9.71)	4F
