# Supplementary material for: Innate immune and proinflammatory signals activate the Hippo pathway via a Tak1-STRIPAK-Tao axis
Source: Nat Commun. 2024 Jan 2;15:145. doi: 10.1038/s41467-023-44542-y (PMC10761881; doi:10.1038/s41467-023-44542-y)
Supplement: Supplementary file 3 — Reporting Summary [file 41467_2023_44542_MOESM3_ESM.pdf]

nature portfolio

Corresponding author(s): Bo Liu

Last updated by author(s): Dec 6, 2023

Reporting Summary

Nature Portfolio wishes to improve the reproducibility of the work that we publish. This form provides structure for consistency and transparency in reporting. For further information on Nature Portfolio policies, see our Editorial Policies and the Editorial Policy Checklist.

Statistics

For all statistical analyses, confirm that the following items are present in the figure legend, table legend, main text, or Methods section.

n/a	Confirmed	
☐	☒	The exact sample size (*n*) for each experimental group/condition, given as a discrete number and unit of measurement
☐	☒	A statement on whether measurements were taken from distinct samples or whether the same sample was measured repeatedly
☐	☒	The statistical test(s) used AND whether they are one- or two-sided<br>*Only common tests should be described solely by name; describe more complex techniques in the Methods section.*
☒	☐	A description of all covariates tested
☐	☒	A description of any assumptions or corrections, such as tests of normality and adjustment for multiple comparisons
☐	☒	A full description of the statistical parameters including central tendency (e.g. means) or other basic estimates (e.g. regression coefficient) AND variation (e.g. standard deviation) or associated estimates of uncertainty (e.g. confidence intervals)
☐	☒	For null hypothesis testing, the test statistic (e.g. *F*, *t*, *r*) with confidence intervals, effect sizes, degrees of freedom and *P* value noted<br>*Give P values as exact values whenever suitable.*
☒	☐	For Bayesian analysis, information on the choice of priors and Markov chain Monte Carlo settings
☒	☐	For hierarchical and complex designs, identification of the appropriate level for tests and full reporting of outcomes
☒	☐	Estimates of effect sizes (e.g. Cohen's *d*, Pearson's *r*), indicating how they were calculated

Our web collection on statistics for biologists contains articles on many of the points above.

Software and code

Policy information about availability of computer code

Data collection	Western blot data was acquired using ChemiDoc Touch Imaging System (Bio-Rad). Immunostaining images were acquired using Zeiss LSM980 +Airyscan2 and ZEISS LSM 780 Laser Scanning Microscope. NovoCyte Quanteon Flow Cytometer Systems (ACEA) was used to acquire Flow Cytometry data, CFX96 real-time system (Bio-Rad) was used to acquire qPCR data and FLUO star Lumiometer (BMG Lab Technologies) was used to acquire luciferase assay data.
Data analysis	Image Lab version 4.1(Rio-Rad) was used to acquire and analyze immunoblots, immunostaining images were analyzed using ZEN Blue 3.1 (Zeiss), Tree Star FlowJo X 10.0.7 R2 was used to analysis Flow cytometry data. All statistical analyses were performed with GraphPad Prism 9.0.1 (GraphPad Software). Quantification of immunoblots was performed using ImageJ (version 1.53t).

For manuscripts utilizing custom algorithms or software that are central to the research but not yet described in published literature, software must be made available to editors and reviewers. We strongly encourage code deposition in a community repository (e.g. GitHub). See the Nature Portfolio guidelines for submitting code & software for further information.

Data

Policy information about availability of data

All manuscripts must include a data availability statement. This statement should provide the following information, where applicable:

- Accession codes, unique identifiers, or web links for publicly available datasets
- A description of any restrictions on data availability
- For clinical datasets or third party data, please ensure that the statement adheres to our policy

All data supporting the findings of this study are available from the corresponding author upon request. The source data for Figs. 1A-K, M, 2, 3A-F, J-L, 4A-D, F-I, 5, 6, 7, and Supplementary Figs. 1, 2, 3A-C, G-J, 4, 5B, 6B, C, 7B, D, 8, 9 are provided as a Source Data file. This paper does not analyze any dataset or report original code. The Drosophila Interaction Database (DroID, http://www.droidb.org/) was used to identify protein interactions. Source data are provided with this paper.

Research involving human participants, their data, or biological material

Policy information about studies with human participants or human data. See also policy information about sex, gender (identity/presentation), and sexual orientation and race, ethnicity and racism.

Reporting on sex and gender	N/A
Reporting on race, ethnicity, or other socially relevant groupings	N/A
Population characteristics	N/A
Recruitment	N/A
Ethics oversight	N/A

Note that full information on the approval of the study protocol must also be provided in the manuscript.

Field-specific reporting

Please select the one below that is the best fit for your research. If you are not sure, read the appropriate sections before making your selection.

☒ Life sciences ☐ Behavioural & social sciences ☐ Ecological, evolutionary & environmental sciences

For a reference copy of the document with all sections, see nature.com/documents/nr-reporting-summary-flat.pdf

Life sciences study design

All studies must disclose on these points even when the disclosure is negative.

Sample size	Sample size was chosen to assure significant statistical differences and the reproducibility of the results. No sample size calculation was performed. Sample size was chosen according to the experiment type and based on what is standard practice in the field (PMID 35905720, 11017107, 26824654, 30119996). Detailed sample size information is provided in the figure legends.
Data exclusions	No data were excluded.
Replication	Experiments were performed in at least three independent biological replicates with similar results.
Randomization	Samples and organisms were randomly allocated to experimental groups. No specific randomization protocol has been used. Fruit flies were age- and sex- matched.
Blinding	No specific blinding was applied since all experiments were assigned into groups including relevant controls and analysis was done objectively and without bias.

Reporting for specific materials, systems and methods

We require information from authors about some types of materials, experimental systems and methods used in many studies. Here, indicate whether each material, system or method listed is relevant to your study. If you are not sure if a list item applies to your research, read the appropriate section before selecting a response.

Materials & experimental systems

n/a	Involved in the study	
☐	☒	Antibodies
☐	☒	Eukaryotic cell lines
☒	☐	Palaeontology and archaeology
☐	☒	Animals and other organisms
☒	☐	Clinical data
☒	☐	Dual use research of concern
☒	☐	Plants

Methods

n/a	Involved in the study	
☒	☐	ChIP-seq
☐	☒	Flow cytometry
☒	☐	MRI-based neuroimaging

Antibodies

Antibodies used

Rabbit anti-β-Actin Cell Signaling Technology Cat# 4967（1：10000）
Mouse anti-HA Sigma-Aldrich Cat# H9658（1：3000）
Rabbit anti-HA Cell Signaling Technology Cat# 3724（1：1000）
Mouse anti-Flag Sigma-Aldrich Cat# F1804（1：5000）
Rat anti-Flag Biolegend Cat# 637304（1：5000）
Mouse anti-c-Myc Sigma-Aldrich Cat# OP10L（1：1000）
Mouse anti-β-galactosidase Developmental Studies Hybridoma Bank Cat# 40-1a（1：100）
Rabbit anti-Thiophosphate ester Abcam Cat# ab133473（1：1000）
Rabbit anti-phospho-Wts T1077 Yu et al., 2010. N/A（1：1000）
Rabbit anti-phospho-Yki S168 Dong et al., 2007. N/A（1：1000）
Rabbit anti-Cka Duojia Pan Lab N/A（1：1000）
Rabbit anti-Hpo Duojia Pan Lab N/A（1：1000）
Rabbit anti-Mats Duojia Pan Lab N/A（1：1000）
Rabbit anti-Slmap Duojia Pan Lab N/A（1：1000）
Rabbit anti-Yki Duojia Pan Lab Dong et al., 2007.（1：1000）
Rabbit anti-phospho-MST1/2 T183/T180 Cell Signaling Technology Cat# 49332（1：1000）
Rabbit anti-phospho-LATS1/2 T1079/T1041 Cell Signaling Technology Cat# 8654（1：1000）
Rabbit anti-phospho-TAOK2 S181 R&D Systems Cat# PPS037（1：1000）
Rabbit anti-Phospho-JNK T183/Y185 Cell Signaling Technology Cat# 4668（1：1000）
Rabbit anti-phospho-YAP S127 Cell Signaling Technology Cat# 4911（1：1000）
Rabbit anti-phospho-MOB T35 Cell Signaling Technology Cat# 3863（1：1000）
Rabbit anti-LATS1 Cell Signaling Technology Cat# 3477（1：1000）
Rabbit anti-LATS2 Cell Signaling Technology Cat# 5888（1：1000）
Mouse anti-YAP Sigma Cat# WH0010413M1（1：1000）
Rabbit anti-STRN ABclonal Cat# A7734（1：1000）
Rabbit anti-GFP Cell Signaling Technology Cat# 2956（1：1000）
Rabbit anti-Cleaved Drosophila Dcp-1 (Asp215) Cell Signaling Technology Cat #9578（1：200）
Goat anti-rat IgG HRP Jackson ImmunoResearch #112-035-003 (1:5000)
Goat anti-rabbit IgG HRP Jackson ImmunoResearch #111-035-003 (1:5000)
Goat anti-mouse IgG HRP Jackson ImmunoResearch #115-035-003 (1:5000)
Goat anti-rabbit IgG Cy3 Jackson ImmunoResearch #111-165-003 (1:500)
Goat anti-rabbit IgG FITC Jackson ImmunoResearch #111-095-003 (1:500)
Goat anti-mouse IgG Cy3 Jackson ImmunoResearch #115-165-003 (1:500)
Goat anti-mouse IgG FITC Jackson ImmunoResearch # 115-095-003 (1:500)

Validation

Rabbit anti-β-Actin Cell Signaling Technology Cat# 4967
https://www.cellsignal.cn/products/primary-antibodies/b-actin-antibody/4967?N=102236+4294956287&Nrpp=30&No=120&fromPage=plp
The manufacturer has validated this antibody for WB in the species human and mouse.
Mouse anti-HA Sigma-Aldrich Cat# H9658
https://www.sigmaaldrich.cn/CN/zh/product/sigma/h9658
The manufacturer has validated this antibody for WB and IP in the species human and mouse.
Rabbit anti-HA Cell Signaling Technology Cat# 3724
https://www.cellsignal.cn/products/primary-antibodies/ha-tag-c29f4-rabbit-mab/3724?site-search-type=Products&N=4294956287&Ntt=%23+3724&fromPage=plp&_requestid=2232722
The manufacturer has validated this antibody for WB, IF and IP in the species human and mouse.
Mouse anti-Flag Sigma-Aldrich Cat# F1804
https://www.sigmaaldrich.cn/CN/zh/product/sigma/f1804
The manufacturer has validated this antibody for WB, IF and IP in the species human and mouse.
Rat anti-Flag Biolegend Cat# 637304
https://www.biolegend.com/en-us/products/purified-anti-dykddddk-tag-antibody-4905

The manufacturer has validated this antibody for WB in the species human.
Mouse anti-c-Myc Sigma-Aldrich Cat# OP10L
https://www.sigmaaldrich.cn/CN/zh/product/mm/op10l
The manufacturer has validated this antibody for WB, IF and IP in the species human and mouse.
Mouse anti-β-galactosidase Developmental Studies Hybridoma Bank Cat# 40-1a
https://dshb.biology.uiowa.edu/40-1a
The manufacturer has validated this antibody for WB, IF and IP.
Rabbit anti-Thiophosphate ester Abcam Cat# ab133473
https://www.abcam.cn/products/primary-antibodies/thiophosphate-ester-antibody-51-8-ab133473.html
The manufacturer has validated this antibody for IP in species independent.
Rabbit anti-phospho-Wts T1077 Yu et al., 2010.
Tao-1 Phosphorylates Hippo/MST kinases to Regulate the Hippo-Salvador-Warts Tumor Suppressor Pathway - PMC (nih.gov)
The antibody has been validated for WB in this paper
Rabbit anti-phospho-Yki S168 Dong et al., 2007.
Elucidation of a Universal Size-Control Mechanism in Drosophila and Mammals - PMC (nih.gov)
The antibody has been validated for WB in this paper
Rabbit anti-Yki Duojia Pan Lab Dong et al., 2007.
Elucidation of a Universal Size-Control Mechanism in Drosophila and Mammals - PMC (nih.gov)
The antibody has been validated for WB and IF in this paper
Rabbit anti-Cka Duojia Pan Lab
Rabbit anti-Slmap Duojia Pan Lab
These two antibodies have been validated for WB in our paper
Rabbit anti-phospho-MST1/2 T183/T180 Cell Signaling Technology Cat# 49332
https://www.cellsignal.cn/products/primary-antibodies/phospho-mst1-thr183-mst2-thr180-e7u1d-rabbit-mab/49332?site-search-type=Products&N=4294956287&Ntt=%23+49332&fromPage=plp&_requestid=2233386
The manufacturer has validated this antibody for WB in the species human and mouse.
Rabbit anti-phospho-LATS1/2 T1079/T1041 Cell Signaling Technology Cat# 8654
https://www.cellsignal.cn/products/primary-antibodies/phospho-lats1-thr1079-d57d3-rabbit-mab/8654?site-search-type=Products&N=4294956287&Ntt=%23+8654&fromPage=plp&_requestid=2179574
The manufacturer has validated this antibody for WB in the species human and mouse.
Rabbit anti-phospho-TAOK2 S181 R&D Systems Cat# PPS037
https://www.rndsystems.com/cn/products/phospho-tao2-s181-antibody_pps037
The manufacturer has validated this antibody for WB in the species human and mouse.
Rabbit anti-Phospho-JNK T183/Y185 Cell Signaling Technology Cat# 4668
https://www.cellsignal.cn/products/primary-antibodies/phospho-sapk-jnk-thr183-tyr185-81e11-rabbit-mab/4668?site-search-type=Products&N=4294956287&Ntt=%23+4668&fromPage=plp&_requestid=2180095
The manufacturer has validated this antibody for WB and IP in the species human and mouse.
Rabbit anti-phospho-YAP S127 Cell Signaling Technology Cat# 4911
https://www.cellsignal.cn/products/primary-antibodies/phospho-yap-ser127-antibody/4911?site-search-type=Products&N=4294956287&Ntt=%23+4911&fromPage=plp&_requestid=2234007
The manufacturer has validated this antibody for WB in the species human and mouse.
Rabbit anti-phospho-MOB T35 Cell Signaling Technology Cat# 3863
https://www.cellsignal.cn/products/primary-antibodies/mob1-antibody/3863?site-search-type=Products&N=4294956287&Ntt=%23+3863&fromPage=plp&_requestid=2180712
The manufacturer has validated this antibody for WB in the species human and mouse.
Rabbit anti-LATS1 Cell Signaling Technology Cat# 3477
https://www.cellsignal.cn/products/primary-antibodies/lats1-c66b5-rabbit-mab/3477?site-search-type=Products&N=4294956287&Ntt=%23+3477&fromPage=plp&_requestid=2181154
The manufacturer has validated this antibody for WB and IP in the species human and mouse.
Rabbit anti-LATS2 Cell Signaling Technology Cat# 5888
https://www.cellsignal.cn/products/primary-antibodies/lats2-d83d6-rabbit-mab/5888?site-search-type=Products&N=4294956287&Ntt=%23+5888&fromPage=plp&_requestid=2234674
The manufacturer has validated this antibody for WB in the species human and mouse.
Mouse anti-YAP Sigma Cat# WH0010413M1
https://www.sigmaaldrich.cn/CN/zh/product/sigma/wh0010413m1
The manufacturer has validated this antibody for WB, IP and IF in the species human.
Rabbit anti-STRN ABclonal Cat# A7734
https://abclonal.com.cn/catalog/A7734
The manufacturer has validated this antibody for WB in the species human and mouse.
Rabbit anti-GFP Cell Signaling Technology Cat# 2956
https://www.cellsignal.cn/products/primary-antibodies/gfp-d5-1-rabbit-mab/2956?site-search-type=Products&N=4294956287&Ntt=%23+2956&fromPage=plp&_requestid=2235296
The manufacturer has validated this antibody for WB in the species human and mouse.
Rabbit anti-Cleaved Drosophila Dcp-1 (Asp215)Cell Signaling Technology Cat #9578
https://www.cellsignal.cn/products/primary-antibodies/cleaved-drosophila-dcp-1-asp215-antibody/9578
The manufacturer has validated this antibody for WB in Drosophila S2 cells.
Goat anti-rat IgG HRP Jackson ImmunoResearch #112-035-003
https://www.jacksonimmuno.com/catalog/products/112-035-003
Goat anti-rabbit IgG HRP Jackson ImmunoResearch #111-035-003

https://www.jacksonimmuno.com/catalog/products/111-035-003
Goat anti-mouse IgG HRP Jackson ImmunoResearch #115-035-003
https://www.jacksonimmuno.com/catalog/products/115-035-003
Goat anti-rabbit IgG Cy3 Jackson ImmunoResearch #111-165-003
https://www.jacksonimmuno.com/catalog/products/111-165-003
Goat anti-rabbit IgG FITC Jackson ImmunoResearch #111-095-003
https://www.jacksonimmuno.com/catalog/products/111-095-003
Goat anti-mouse IgG Cy3 Jackson ImmunoResearch #115-165-003
https://www.jacksonimmuno.com/catalog/products/115-165-003
Goat anti-mouse IgG FITC Jackson ImmunoResearch # 115-095-003
https://www.jacksonimmuno.com/catalog/products/115-095-003

Eukaryotic cell lines

Policy information about cell lines and Sex and Gender in Research

Cell line source(s)	D. melanogaster S2R+ cells (FlyBase: FBtc0000150, RRID: CVCL_Z831), D. melanogaster S2 cells (FlyBase: FBtc0000006, RRID: CVCL_TZ72), Human HEK293T cells (ATCC CRL-11268, RRID: CVCL_1926), Human THP-1 cells (ATCC TIB-202, RRID: CVCL_0006), Mouse MEF cells (ATCC CRL-2991, RRID:CVCL_L690).
Authentication	Authentication was not performed for this study. They were bought directly from ATCC and DGRC. Morphological shape of cell lines was monitored during the whole experimental process.
Mycoplasma contamination	All cell lines were tested negative for mycoplasma contamination.
Commonly misidentified lines (See ICLAC register)	No commonly misidentified cell lines were used.

Animals and other research organisms

Policy information about studies involving animals; ARRIVE guidelines recommended for reporting animal research, and Sex and Gender in Research

Laboratory animals	Drosophila were raised on standard cornmeal-agar medium (around 25°C with 40% humidity and 12-h dark/light cycle). Stag 17 embryos, third instar larvae, 3- to 6-days old adults were used in this study. w1118 flies were used as a standard wild-type strain. Hml-Gal4 (stock ID 30141), Hml>GFP (stock ID 30142), en-Gal4 (stock ID 83350), sev-Gal4 (stock ID 5793), UAS-Tak1 (stock ID 58810), Tak1-/- (stock ID 26272), Atg1 RNAi (stock ID 26731), Atg101 RNAi (stock ID 34360) and ban-lacZ (stock ID 10154) lines were obtained from Bloomington Drosophila Stock Center (BDSC). Tak1 RNAi (stock ID 101357), Tao-1 RNAi (stock ID 17432), Hpo RNAi (stock ID 104169), Wts RNAi (stock ID 106174), Atg13 RNAi (stock ID 27955) and Yki RNAi (stock ID 40497) lines were collected from Vienna Drosophila Resource Center (VDRC). Atg9 RNAi (stock ID THU2895) was collected from TsingHua Fly Center. UAS-Yki line has been reported previously. (PMID: 16096061)
Wild animals	This study did not involve the wild animals.
Reporting on sex	Adult male flies, aged 3-6 days were selected for bacteria infection and survival experiments.
Field-collected samples	This study did not involve field-collected samples.
Ethics oversight	No vertebrate animals are involved in this study.

Note that full information on the approval of the study protocol must also be provided in the manuscript.

Flow Cytometry

Plots

Confirm that:

- [x] The axis labels state the marker and fluorochrome used (e.g. CD4-FITC).
- [x] The axis scales are clearly visible. Include numbers along axes only for bottom left plot of group (a 'group' is an analysis of identical markers).
- [x] All plots are contour plots with outliers or pseudocolor plots.
- [x] A numerical value for number of cells or percentage (with statistics) is provided.

Methodology

Sample preparation	Flow Cytometry analysis was performed in the primary hemocytes isolated from 3rd instar larvae, detailed information was reported in the Methods section.

Instrument	NovoCyte Quanteon Flow Cytometer Systems (ACEA)
Software	Tree Star FlowJo X 10.0.7 R2
Cell population abundance	Moderate
Gating strategy	In our study, hemocytes were circled as the E1 gate. Then, FITC was used to gate cells that have engulfed GFP-positive E. coli.

☒ Tick this box to confirm that a figure exemplifying the gating strategy is provided in the Supplementary Information.
